# Psychosocial Factors Affecting Artificial Intelligence Adoption in Health Care in China: Cross-Sectional Study

**DOI:** 10.2196/14316

**Published:** 2019-10-17

**Authors:** Tiantian Ye, Jiaolong Xue, Mingguang He, Jing Gu, Haotian Lin, Bin Xu, Yu Cheng

**Affiliations:** 1 Department of Anthropology School of Sociology and Anthropology Sun Yat-sen University Guangzhou China; 2 Department of Preventive Ophthalmology Zhongshan Ophthalmic Center Sun Yat-sen University Guangzhou China; 3 Business School Sun Yat-sen University Guangzhou China; 4 School of Management Guangdong Ocean University Zhanjiang China; 5 State Key Laboratory of Ophthalmology Zhongshan Ophthalmic Center Sun Yat-sen University Guangzhou China; 6 Center for Eye Research Australia University of Melbourne Melbourne Australia; 7 Department of Epidemiology and Medical Statistics School of Public Health Sun Yat-sen University Guangzhou China; 8 Guangzhou Yuexiu Center for Disease Control and Prevention Guangzhou China; 9 Department of Medical Humanities The Seventh Affiliated Hospital Sun Yat-sen University Shenzhen China

**Keywords:** artificial intelligence, adoption, technology acceptance model, structural equation model, intention, subjective norms, trust, moderation

## Abstract

**Background:**

Poor quality primary health care is a major issue in China, particularly in blindness prevention. Artificial intelligence (AI) could provide early screening and accurate auxiliary diagnosis to improve primary care services and reduce unnecessary referrals, but the application of AI in medical settings is still an emerging field.

**Objective:**

This study aimed to investigate the general public’s acceptance of ophthalmic AI devices, with reference to those already used in China, and the interrelated influencing factors that shape people’s intention to use these devices.

**Methods:**

We proposed a model of ophthalmic AI acceptance based on technology acceptance theories and variables from other health care–related studies. The model was verified via a 32-item questionnaire with 7-point Likert scales completed by 474 respondents (nationally random sampled). Structural equation modeling was used to evaluate item and construct reliability and validity via a confirmatory factor analysis, and the model’s path effects, significance, goodness of fit, and mediation and moderation effects were analyzed.

**Results:**

Standardized factor loadings of items were between 0.583 and 0.876. Composite reliability of 9 constructs ranged from 0.673 to 0.841. The discriminant validity of all constructs met the Fornell and Larcker criteria. Model fit indicators such as standardized root mean square residual (0.057), comparative fit index (0.915), and root mean squared error of approximation (0.049) demonstrated good fit. Intention to use (R^2^=0.515) is significantly affected by subjective norms (beta=.408; *P*<.001), perceived usefulness (beta=.336; *P*=.03), and resistance bias (beta=–.237; *P*=.02). Subjective norms and perceived behavior control had an indirect impact on intention to use through perceived usefulness and perceived ease of use. Eye health consciousness had an indirect positive effect on intention to use through perceived usefulness. Trust had a significant moderation effect (beta=–.095; *P*=.049) on the effect path of perceived usefulness to intention to use.

**Conclusions:**

The item, construct, and model indicators indicate reliable interpretation power and help explain the levels of public acceptance of ophthalmic AI devices in China. The influence of subjective norms can be linked to Confucian culture, collectivism, authoritarianism, and conformity mentality in China. Overall, the use of AI in diagnostics and clinical laboratory analysis is underdeveloped, and the Chinese public are generally mistrustful of medical staff and the Chinese medical system. Stakeholders such as doctors and AI suppliers should therefore avoid making misleading or over-exaggerated claims in the promotion of AI health care products.

## Introduction

### Background

As part of the fourth industrial revolution, artificial intelligence (AI) has achieved massive progress and explosive growth. It is actively applied in health care to perform a wide range of functions such as patient administration and monitoring, clinical decision support, risk prediction, medical error reduction, health care intervention, and productivity improvement [1,2]. These potential benefits could contribute greatly to primary care services in China, where the health system is facing great challenges owing to an aging population and an increase in chronic noncommunicable diseases [3].

This challenge is especially crucial for eye health management in China, where rates of blindness and vision impairment are the highest in the world and age-related eye diseases such as cataracts, diabetic retinopathy (DR), and juvenile myopia are increasingly common [4]. Most of these diseases cannot be diagnosed in primary care institutions, so patients seek direct care from ophthalmologists in tertiary hospitals without a referral. Data from 1 survey in Shanghai showed that on average there are only 0.09 ophthalmologists and 0.1 primary eye care (PEC) providers for every 10,000 people [5]. Of the available ophthalmologists and PEC providers, 82.9% majored in public health, nursing, or internal medicine and have not had specialist ophthalmic training. The situation is even worse in areas of western China, such as Tibet and Inner Mongolia, where the high prevalence of blindness and poor vision has become a serious public health issue. It is vital to establish and maintain an appropriate, effective eye care program in these areas [6,7].

Researchers have demonstrated that the performance of image-based AI devices can reach or even surpass that of experts [8-10]. The number of effective programs and policies to prevent blindness in China has increased [11], and a number of ophthalmic AI devices are available in clinical scenarios, such as EyeGrader (Center for Eye Research Australia, Melbourne, Australia) for the detection of DR [12] and CC-Cruiser (Zhongshan Ophthalmic Center, Guangzhou, China) for congenital cataracts [13]. Stakeholders such as doctors and AI suppliers are trying to apply these devices in clinical settings such as health check centers, community health centers (CHCs), schools, optical stores, and grassroots hospitals in rural China [14]. As no prior studies have been conducted on the implementation of ophthalmic AI devices in the Chinese context, we have briefly described the results of our formative qualitative studies of 3 CHCs where an ophthalmic AI device was used (unpublished). During the implementation period from April 1 to December 31, 2018, the total number of people who signed the Service of Community Family Physician was 63,034. We found that the low number of patients who chose to receive AI screening (3067 out of 63,034) could reflect public unwillingness to use these devices, though AI screening was not systematically offered by physicians. In interviews with patients, we found that patients were unwilling to undergo this process unless it was provided free of charge, arranged by their work unit, or they could attend the screening in a group with other people.

Above all, in recent years, AI health care researchers have focused on technical innovation and clinical results, without considering the human context or ethical challenges that are invariably involved in any complex health care system. Many real-world issues need to be assessed in the implementation phase, most notably the extent to which patients or the public accept AI and the challenges involved in protecting patient privacy and confidential medical information. Thus, understanding the factors that influence public acceptance of (or resistance to) AI devices in the Chinese social and cultural context will help government agencies and health care administrators to devise appropriate intervention strategies to minimize user resistance and its negative effects on health care policy.

### Objective

The aims of this study were to develop and test a model investigating the factors that drive the public’s acceptance of ophthalmic AI devices, with reference to those already used in primary care institutions in China. In particular, we aimed to evaluate how subjective norms, resistance bias, and trust contribute to the relationships among these factors in the Chinese cultural context.

### Theoretical Background and Hypothesis Development

Many technology adoption models have been proposed to explain user adoption of new technology and to assess the factors that can affect user acceptance [15]; examples include the Technology Acceptance Model (TAM) [16,17], Theory of Planned Behavior (TPB) [18,19], and the Unified Theory of Acceptance and Use of Technology (UTAUT) [20]. Many medical information researchers have modified and combined models or added new constructs to carry out studies in domains such as telemedicine [21-23], clinical decision systems [24,25], electronic health care records [26-29], mobile medical information systems [30-32], and personal digital assistants [33-35].

Studies of the acceptance of new health care technology have identified influential factors and reliable correlations between those factors and the acceptance or usage of new technology. However, very few studies have been carried out in relation to AI technology. As ophthalmic AI devices are an emerging technology, this study uses the following theories and constructs to evaluate these influential factors and facilitate the application of AI within primary health care institutions.

#### Technology Acceptance Model Theories

The TAM is the most widely applied model to describe consumer acceptability of information technology [36]. The original model, developed by Fred D Davis in 1989 [16], revealed that perceived usefulness (PU; defined as the perception that using a system leads to enhanced job performance) and perceived ease of use (PEOU; defined as the perception that using a system will be free of effort) were 2 basic determinants of people’s acceptance of new technology, which is now commonly evaluated by behavioral intention to use (IU; defined as an individual’s motivation or willingness to exert effort to perform the target behavior) [15,16]. Then on, many researchers have added, modified, or deleted some variables to synthesize new models to fit their studies, such as TAM 2, TAM 3, and UTAUT. However, many researchers found that both PU and PEOU had a direct effect on IU without a mediation effect of attitude, and attitude was deleted in the following TAMs [36,37]. In our study, although the purpose was to understand the Chinese public’s acceptance of AI devices, as most regions do not have access to these devices, our final dependent variable was IU, rather than actual usage behavior as indicated in the TAMs.

IU is now commonly used to refer to acceptance and is considered to reliably predict actual use; it is sometimes the only measured outcome of interest in TAM-related studies [15]. Studies have shown that PU and PEOU exert considerable positive influence on IU, and PEOU has an effect on PU [15,16]. We thus proposed the following hypotheses:

- H1: Perceived usefulness positively affects the public’s intention to use ophthalmic AI devices.
- H2a: Perceived ease of use positively affects the public’s intention to use ophthalmic AI devices.
- H2b: Perceived ease of use positively affects the public’s perception of the usefulness of ophthalmic AI devices.

#### Theory of Planned Behavior

The Theory of Planned Behavior (TPB) states that an individual’s behavioral intention (similar to IU in TAMs) is determined by attitude, perceived behavioral control (PBC; the extent to which people have control over engaging in the behavior) and subjective norms (SN; defined as perceptions of whether others think one should engage in a behavior) [38-40]. TPB, a more comprehensive version of the Theory of Reasoned Action (TRA) [41], allows us to examine the influence of personal determinants and social surroundings as well as nonvolitional determinants on IU [42]. As an extension of the TRA, TPB has been one of the most widely tested models of the factors influencing health-related behavior [40]. SN has a direct effect on IU in the UTAUT and TPB models and an indirect impact on IU through PEOU in many integrated models [15,36,43]. PBC has a positive effect on IU in the TPB and UTAUT models [15,44]. However, when combined with TAMs, PBC also has an indirect effect through PEOU [45,46]. Therefore, we proposed the following integrated hypotheses:

- H3a: Subjective norms positively affect the public’s intention to use ophthalmic AI devices directly.
- H3b: Subjective norms positively affect the public’s perception of the ease of use of ophthalmic AI devices.
- H4a: Perceived behavioral control positively affects the public’s intention to use ophthalmic AI devices directly.
- H4b: Perceived behavioral control positively affects the public’s perception of the ease of use of ophthalmic AI devices.

#### Health Belief Model and Eye Health Consciousness

The health belief model (HBM) [47] was initially designed to “understand the widespread failure of people to accept preventives or screening tests for the early detection of asymptomatic disease” [48]. In later studies, the HBM was used to predict more general health-related behaviors, to understand why individuals did or did not engage in these actions, and to explain and predict the acceptance of health and medical care recommendations [36,48,49]. Health consciousness is defined as the “degree to which health concerns are integrated into a person’s daily activities and health-conscious people are aware of and concerned about their wellness, resulting in a better motivation to improve or maintain their health” [49]. Health beliefs and concerns have an indirect effect on behavioral intention to use health information technology via the remote mediation effect of perceived health threat (PHT) and PU [36]. One study in China examined patients’ acceptance of mobile phone health technology for chronic disease management and showed that PHT had a significant positive effect on PU together with a positive effect directly on IU [31]. In that study, PHT referred to patients’ awareness and care of the health condition and its potential consequences. The items in their construct also covered a person’s degree of consciousness, beliefs, and awareness of hypertension and health management and asked the participants if they were aware of or concerned about blood pressure and would make efforts to manage hypertension. Therefore, we modified these items to fit the eye care context and defined this construct as eye health consciousness (EHC). We thus proposed the following hypothesis:

- H5a: Eye health consciousness positively affects the public’s intention to use ophthalmic AI devices directly.
- H5b: Eye health consciousness positively affects the public’s perception of the usefulness of ophthalmic AI devices directly.

#### Dual Factor Theory and Status Quo Bias Theory

The above health behavior theories focus almost exclusively on users’ positive (enabling) perceptions in relation to new technology usage and ignore negative (inhibiting) factors [31,50]. However, in the Dual Factor Theory (DFT), potential users’ information technology usage considerations are based on a simultaneous examination of both enabling and inhibiting factors [31]. Inhibitors discourage information systems (IS) usage when present but do not necessarily favor usage when absent. They are not quite the opposite of enablers but are qualitatively distinct constructs that are independent of but may coexist with enablers [51]. Perceived risk (PR) refers to the combination of uncertainty and the seriousness of an outcome in relation to performance, safety, and psychological or social uncertainties, which have a negative influence on IU and are thus barriers to adoption [28,52,53]. Status Quo Bias (SQB) theory aims to explain people’s preference for maintaining their current status or situation and provides a set of useful theoretical explanations for understanding the impact of incumbent system (IS) use as an inhibitor of new IS acceptance. For example, data on the selection of health plans by faculty members reveal that SQB is substantial in important real-world decisions [54], so several studies have modified their models by supplementing the negative (inhibiting) constructs of SQB theory with user resistance factors that are a type of inhibitor [51,55]. The 2 main inhibitors are regret avoidance (lessons from experiences that have taught individuals to avoid regrettable consequences) and inertia (an individual’s attachment to his or her current situation even if there are better alternatives or incentives to change) [51,55]. Resistance to change (RTC) refers to people’s attempts to maintain their previous behaviors or habits that are connected to their past experiences when facing change [31,56-58]. RTC has been confirmed as a major barrier for electronic health and mobile health adoption [56-58]. We integrated these factors into 1 inhibitor, resistance bias (RB), defined as people’s resistance to use a new technology owing to biases such as regret avoidance, inertia, and RTC. We thus proposed the following hypothesis:

- H6: Perceived risk negatively affects the public’s intention to use ophthalmic AI devices.
- H7: Resistance bias negatively affects the public’s intention to use ophthalmic AI devices.

#### Trust as a Moderator in the Chinese Social Context

Trust is defined as the belief that someone or something is honest, reliable, good, and effective, or the desire to depend on someone or something for security [44]. Various studies show that it has a direct or indirect mediation effect on user intention or adoption of new technology [52,59-61]. With the increasing proliferation of AI applications in daily life, consideration of trust is essential because it is likely to be a critical factor in the acceptance of consumer products such as home automation, personal robots, and automotive automation [62,63]. Moreover, acceptance behaviors for technologies are controlled and moderated by cultural traits [64,65].

In China, patients’ trust of physicians is lower than in Western countries [66] and has become a serious social problem [67,68]. Trust in applied AI is an evolving phenomenon, and cognitive compatibility, trialability, and usability are the main factors related to trust in a technology [63]. The public’s trust might play a more complicated role in relation to AI devices in China, affecting the factors that influence IU. In the field of health care research, no previous studies have tested trust of the public as a moderator between PU and IU in China. However, Cuadrado identified the moderating effects of trust, showing that trust levels strengthened the negative effect of prosocialness on selfish irrigation strategies [69]. Although irrigation is unrelated to health or AI, it provides evidence and the possibility that trust might have a potential moderating effect in our context. Thus, we proposed a new hypothesis:

- H8: Trust of physicians moderates the effect of perceived usefulness on the public’s intention to use ophthalmic AI devices.

**Figure 1:**
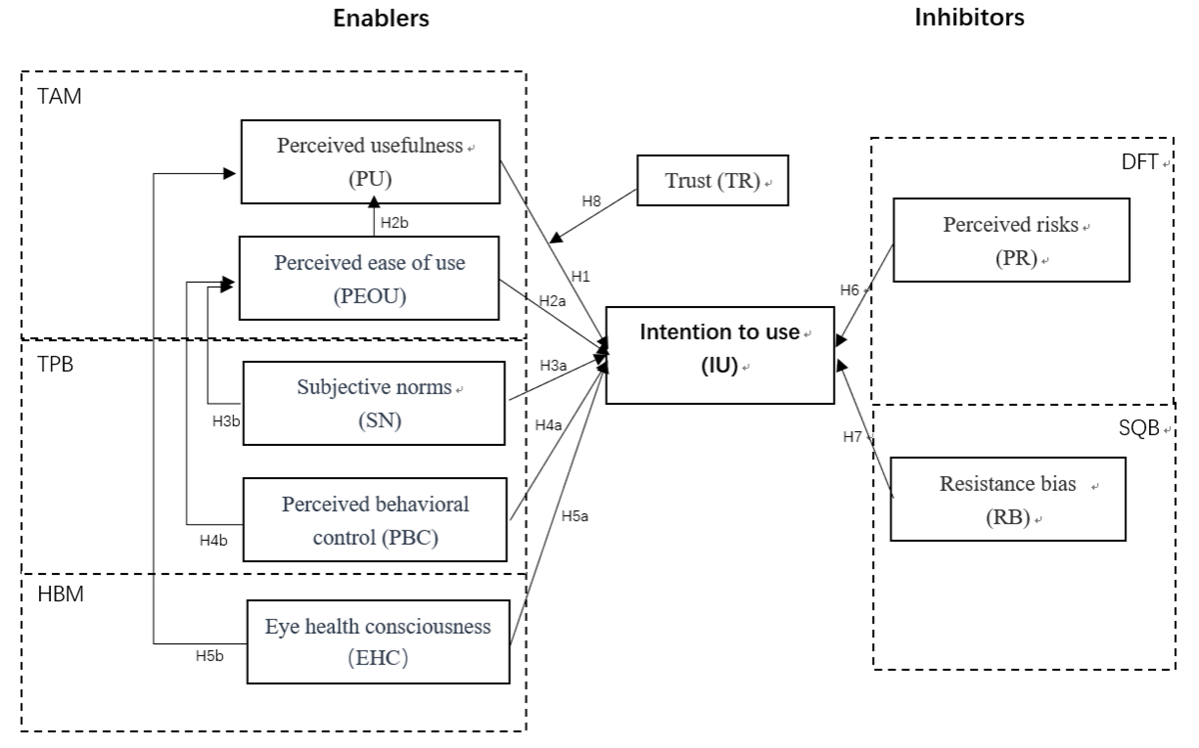
Variables from relevant theories and development of our model for ophthalmic artificial intelligence device acceptance. DFT: Dual Factor Theory; HBM: health belief model; SQB: status quo bias; TAM: Technology Acceptance Model; TPB: Theory of Planned Behavior.

Overall, this study proposes and evaluates 12 hypotheses with TAM and TPB as the underpinning theories (Figure 1). We added the constructs EHC, PR, and RB from HBM, DFT, and SQB, respectively, to fit our context as these constructs have been validated in the previous studies in China or other parts of Asia. Trust was also added as a moderator to reflect the significance of physician-patient relationships in the Chinese context. The selection of variables from relevant theories and the development of our model are shown in Figure 1.

## Methods

### Participants and Sampling

Potential end users of ophthalmic AI devices in China were recruited if they (1) resided in China (including both urban and rural areas of different provinces and people in all age groups and career types); (2) could read and write in Chinese; (3) had a mobile phone or sufficient internet access; and (4) were not ophthalmic medical staff such as ophthalmologists or nurses.

On the basis of these criteria, we worked with a Web-based company to recruit participants. We calculated the required number of participants based on a sample size rule of thumb for structural equation modeling of 10 times the number of participants as items [70]. As our survey had 32 items, the required number of participants was more than 320. The company distributed the survey to 925 potential participants from January 20 to 24, 2019. The company used simple random sampling of people who were mobile phone users. Its sample database was our source for randomly sampling, which has more than 2.6 million members. Technicians sent selected participants a direct message over a popular messaging platform (WeChat) with the link to the invitation of our questionnaire during certain times on data collection days. Surveys could be completed by potential participants using WeChat. The survey company’s website [71] showed the information about the sample source, which was verified and randomized with different job categories. Every day, more than one million people answered questionnaires on this survey platform.

The criteria for determining the completeness of a questionnaire included (1) each account responded only once; (2) the response time was longer than 300 seconds to exclude perfunctory respondents; (3) one *identifying item* randomly selected from an *item bank*, such as *please select the right alphabetical sequence of the following letters: bcdefg*, had to be answered correctly; and (4) anyone choosing *ophthalmic medical staff* in the final *identification item* was excluded.

### Measurement

The 9 constructs in the hypothesis model were measured by 32 questionnaire items. Each item measured only 1 construct (variable or factor). All items were sourced from the relevant literature related to consumer technology acceptance research, with some changes to fit the ophthalmic AI context (Table 1). Items in English were translated into Chinese by 1 researcher and checked by 3 other researchers, and 1 researcher then back translated the items into English to check if the original meaning was retained. All researchers are bilingual fluent in English and Chinese. All items were measured on 7-point Likert scales ranging from (1) strongly disagree to (7) strongly agree.

**Table 1:** Constructs, items, and references of the measurements.

Construct		Definition and items	References
**Perceived usefulness (PU)**		The degree to which a person believes that the use of ophthalmic AI^a^ devices would enhance his or her personal or job performance	[15,16,31,72,73]
	PU1	Ophthalmic AI devices would help me to cope with preventable eye diseases at an early stage	[16,31]
	PU2	Ophthalmic AI devices would provide detailed information and images of my eyes, which would be very useful for me	[16,31]
	PU3	Ophthalmic AI devices would help the medical institutions to recognize more treatable eye patients	[16,31]
	PU4	Ophthalmic AI devices would improve primary health care for health departments and save money	[16,31]
	PU5	Ophthalmic AI devices would be a good supplement to traditional health care approaches and fit with my medical philosophy	[16,31]
	PU6	Ophthalmic AI devices would fit my demand for eye health management	[16,31]
	PU7	Ophthalmic AI devices would achieve the same results as face-to-face diagnosis with an ophthalmologist	[16,31]
**Perceived ease of use (PEOU)**		The degree to which a person believes that ophthalmic AI devices would be easy to use	[15,16,31,72]
	PEOU1	I find the instructions for ophthalmic AI devices easy, clear, and understandable	[16,31]
	PEOU2	Ophthalmic AI devices would offer a more convenient way for me to cope with my eye disease without queuing for registration in hospitals and would save me time and money	[16,31]
**Perceived behavioral control (PBC)**		Perception of internal and external resource constraints to using ophthalmic AI devices, or the availability of skills, resources, and opportunities necessary to use them	[15,18,32,44]
	PBC1	I have enough knowledge to recognize whether the results of the report are reliable	[15,44]
	PBC2	I would receive appropriate technical assistance when encountering any difficulties in using ophthalmic AI devices or understanding the report	[15,44]
	PBC3	I would be able to use ophthalmic AI devices independently as long as I had enough time and made an effort to learn	[15,44]
**Subjective norms (SN)**		Perception of important (or relevant) others’ beliefs about my use of ophthalmic AI devices	[15,18,43,44,52]
	SN1	People who are important to me (family members, relatives, and close friends) think that I should use ophthalmic AI devices	[15,44]
	SN2	My colleagues or peers think that I should use ophthalmic AI devices	[15,44]
	SN3	My leaders or superiors think that I should use ophthalmic AI devices	[15,44]
**Trust (TR)**		The extent to which an individual believes that using ophthalmic AI devices is secure, reliable, effective, and poses no privacy threats	[44,52]
	TR1	I would trust that with big data and deep learning, ophthalmic AI devices could deliver a reliable report after analyzing my eye health images	[44,52]
	TR2	I would trust that ophthalmic AI devices are more accurate and reliable than human ophthalmologists, because they do not make subjective or empirical errors	[44,52]
	TR3	I would trust that stakeholders and reliable third parties would ensure the security and privacy of my personal data, health information, and images	[44,52]
**Resistance bias (RB)**		Resistance to a new technology owing to biases such as regret avoidance, inertia, and resistance to change	[31,51,56-58]
	RB1	I don’t want ophthalmic AI devices to change how I deal with eye diseases because I can’t be bothered and they are unfamiliar to me	[31,51]
	RB2	I don’t want to use ophthalmic AI devices because from past experience, these new high-tech products always fall flat during practical applications	[31,51]
	RB3	I might regret trying to use these ophthalmic devices because they could waste my time and effort	[31,51]
**Eye health consciousness (EHC)**		Awareness and care of eye health conditions, and the degree to which eye health concerns are integrated into a person’s daily activities	[31,49]
	EHC1	I am aware of and very concerned about my eye health	[31,49]
	EHC2	I would make efforts to manage my eye health	[31,49]
**Perceived risks (PR)**		A combination of uncertainty and seriousness of an outcome in relation to performance, safety, psychological or social uncertainties	[28,52,53,73]
	PR1	There is a possibility of malfunction and performance failure, so they might fail to deliver accurate diagnoses or recommendations and could increase conflicts between members of the public and medical institutions	[52,53]
	PR2	I am concerned that my personal information and health details would be insecure and could be accessed by stakeholders or unauthorized persons, leading to misuse and discrimination	[52,53]
	PR3	Considering the difficulties involved in taking high-quality images for AI analysis, I think there is a risk of incorrect screening results	[52,53]
	PR4	Given the vision problems I possibly already have, such as visual fatigue, dry eye, or presbyopia, I might find it hard to read the printed or electronic report from ophthalmic AI devices	[52,53]
	PR5	Because I might have difficulty understanding the screening report correctly by myself, it might increase my anxiety about my eye health	[52,53]
	PR6	Because practitioners with little ophthalmic knowledge might find it difficult to understand the screening report and explain the terminology and results to me, they might increase my anxiety of about my eye health	[52,53]
**Intention to use (IU)**		An individual’s motivation or willingness to exert effort to use ophthalmic AI devices	[15,43,44]
	IU1	I intend to use ophthalmic AI devices as my first choice if I feel eye discomfort	[15,44]
	IU2	I will encourage my friends/relatives to use ophthalmic AI devices first if they feel eye discomfort	[15,44]
	IU3	I will encourage healthy people to use ophthalmic AI devices for eye health path screening	[15,44]

^a^AI: artificial intelligence.

The first page of the questionnaire provided an overview of the study background, purpose, voluntary nature, and anonymity, and asked respondents to indicate their consent. The participants were assured that the questionnaires would only be used by the researchers and would not be accessible to anyone else. On the second page of the questionnaire, we provided a brief introduction to ophthalmic AI devices, including their general functions and operating procedures, with photographs to help instruct the participants. Table 1 shows the constructs and items of the questionnaire and the literature references. We paid ¥12 (US $1.5) to the survey company for each of the 474 completed questionnaires. The company then paid each participant ¥4 (US $0.5). Our Web-based survey was in accordance with the required Checklist for Reporting Results of Internet E-Surveys (Multimedia Appendix 1). Ethical approval was obtained from the Ethics Committee of the Zhongshan Ophthalmic Center, Sun Yat-Sen University.

### Data Analysis

SPSS version 25.0 was used to analyze the descriptive statistics. Model evaluation involved a 2-step analysis [74] using Amos 21.0 software by (1) evaluating item and construct reliability and validity via confirmatory factor analysis of the measurement model and (2) evaluating the structural model’s path effects, significance, and goodness of fit and mediation and moderation effects.

## Results

### Demographic Results

We distributed Web-based surveys to 925 potential participants, and 732 individuals participated in the survey (rate of participation, 79.1%, 732/925). Of these, 474 (rate of completion, 64.8%, 474/732) participants who completed the questionnaire and met the criteria were used for the SEM analysis. The participants’ demographic characteristics are represented in Table 2. The participants’ geographical origins are shown in Table 3.

**Table 2:** Demographic results.

Characteristics			Values, n (%)
**Gender**			
	Male	169 (35.7)	
	Female	305 (64.3)	
**Age (years)**			
	<18	3 (0.6)	
	18-25	128 (27.0)	
	26-30	132 (27.8)	
	31-40	175 (36.9)	
	41-50	23 (4.9)	
	51-60	11 (2.3)	
	>60	2 (0.4)	
**Education**			
	Middle school	4 (0.8)	
	High school	8 (1.7)	
	Three-year college	64 (13.5)	
	Bachelor’s degree	341 (71.9)	
	Master’s degree	54 (11.4)	
	Doctoral degree	3(0.6)	

**Table 3:** Geographical origins of participants (N=474).

Province	Value, n (%)
Guangdong	80 (16.9)
Beijing	67 (14.1)
Shanghai	38 (8.0)
Jiangsu	37 (7.8)
Shandong	28 (5.9)
Zhejiang	26 (5.5)
Sichuan	22 (4.6)
Henan	17 (3.6)
Hubei	17 (3.6)
Liaoning	17 (3.6)
Chongqing	16 (3.4)
Anhui	15 (3.2)
Hunan	13 (2.7)
Shaanxi	13 (2.7)
Hebei	10 (2.1)
Fujian	9 (1.9)
Heilongjiang	8 (1.7)
Jiangxi	8 (1.7)
Shanxi	8 (1.7)
Jilin	5 (1.1)
Tianjin	5 (1.1)
Guangxi	4 (0.8)
Yunnan	4 (0.8)
Guizhou	2 (0.4)
Gansu	1 (0.2)
Hainan	1 (0.2)
Inner Mongolia	1 (0.2)
Ningxia	1 (0.2)
Xinjiang	1 (0.2)

### The Effect of Education on Intention to Use

The results of a single-factor analysis of variance showed that the main effect of education on IU was not significant (*F*_5,468_=0.316; *P*>.05) and that each group of education had no significant difference in terms of IU, with means from 4.750 to 5.204, as shown in Table 4. As predicted, the results of post hoc comparisons revealed that the effect of education on IU was not significant as shown in Table 5.

**Table 4:** Descriptive statistics of the effect of education on intention to use.

Diploma	Total	Mean (SD)	SE	95% CI for mean	Minimum	Maximum
Middle school	4	4.750 (1.912)	0.956	1.707 to 7.793	3.000	7.000
High school	8	5.375 (1.408)	0.498	4.198 to 6.552	2.333	6.667
Three-year college	64	5.167 (0.914)	0.114	4.938 to 5.395	2.333	7.000
Bachelor’s degree	341	5.199 (1.000)	0.054	5.093 to 5.306	1.000	7.000
Master’s degree	54	5.204 (1.084)	0.148	4.908 to 5.500	2.333	6.667
Doctoral degree	3	4.778 (1.347)	0.778	1.431 to 8.124	3.333	6.000

**Table 5:** Post hoc multiple comparisons of the effect of education on intention to use (IU; dependent variable: IU Method: Scheffe).

Diploma (I), diploma (J)		Mean difference (I-J)	SE	*P* value	95% CI
**Middle school**					
	High school	–0.625	0.622	.96	–2.704 to 1.454
	Three-year college	–0.417	0.524	.99	–2.167 to 1.333
	Bachelor’s degree	–0.449	0.511	.98	–2.157 to 1.258
	Master’s degree	–0.454	0.527	.98	–2.213 to 1.306
	Doctoral degree	–0.028	0.776	>.99	–2.621 to 2.566
**High school**					
	Middle school	0.625	0.622	.96	–1.454 to 2.704
	Three-year college	0.208	0.381	>.99	–1.065 to 1.482
	Bachelor’s degree	0.176	0.363	>.99	–1.039 to 1.390
	Master’s degree	0.171	0.385	>.99	–1.115 to 1.458
	Doctoral degree	0.597	0.688	.98	–1.702 to 2.896
**Three-year college**					
	Middle school	0.417	0.524	.99	–1.333 to 2.167
	High school	–0.208	0.381	>.99	–1.482 to 1.065
	Bachelor’s degree	–0.033	0.138	>.99	–.495 to .430
	Master’s degree	–0.037	0.188	>.99	–.664 to .590
	Doctoral degree	0.389	0.600	>.99	–1.617 to 2.395
**Bachelor’s degree**					
	Middle school	0.449	0.511	.98	–1.258 to 2.157
	High school	–0.176	0.363	>.99	–1.390 to 1.039
	Three-year college	0.033	0.138	>.99	–.430 to .495
	Master’s degree	–0.004	0.149	>.99	–.502 to .493
	Doctoral degree	0.422	0.589	>.99	–1.547 to 2.391
**Master’s degree**					
	Middle school	0.454	0.527	.98	–1.306 to 2.213
	High school	–0.171	0.385	>.99	–1.458 to 1.115
	Three-year college	0.037	0.188	>.99	–.590 to .664
	Bachelor’s degree	0.004	0.149	>.99	–.493 to .502
	Doctoral degree	0.426	0.603	>.99	–1.588 to 2.440
**Doctoral degree**					
	Middle school	0.028	0.776	>.99	–2.566 to 2.621
	High school	–0.597	0.688	.98	–2.896 to 1.702
	Three-year college	–0.389	0.600	>.99	–2.395 to 1.617
	Bachelor’s degree	–0.422	0.589	>.99	–2.391 to 1.547
	Master’s degree	–0.426	0.603	>.99	–2.440 to 1.588

### Measurement Model

Maximum likelihood estimation was used to test the factor loadings, measurement reliability, convergent validity, and discriminant validity. Table 6 presents a summary of the significance tests, item reliability, composite reliability (CR), and convergence validity. The standardized factor loadings of items are between 0.583 and 0.869, with good item reliability. The CR values of the 9 constructs range from 0.673 to 0.841, approaching or exceeding 0.7 [75] All constructs have acceptable internal consistency. Most constructs have an average variance extracted (AVE) value higher than the threshold of 0.5, which confirms the constructs’ convergent validity.

**Table 6:** Descriptive statistics of variables, items, and convergent validity.

Construct, item		Mean	Significant test of parameter estimation				Item reliability		Composite reliability, CR^d^	Convergence validity, AVE^e^
			Unstd^a^	SE	Unstd/SE	*P* value	STD^b^	SMC^c^		
**Perceived usefulness (PU)**									0.841	0.431
	PU1	6.095	1	—^f^	—	—	0.663	0.44		
	PU2	6.171	1.076	0.09	11.972	<.001	0.638	0.407		
	PU3	6.118	1.118	0.094	11.926	<.001	0.629	0.396		
	PU4	5.859	1.344	0.118	11.386	<.001	0.605	0.366		
	PU5	5.873	1.419	0.116	12.222	<.001	0.656	0.43		
	PU6	5.77	1.518	0.115	13.149	<.001	0.727	0.529		
	PU7	5.091	1.958	0.156	12.512	<.001	0.672	0.452		
**Perceived ease of use (PEOU)**									0.68	0.516
	PEOU1	5.715	1	—	—	—	0.685	0.469		
	PEOU2	5.762	1.109	0.119	9.313	<.001	0.75	0.562		
**Perceived behavioral control (PBC)**									0.673	0.408
	PBC1	4.62	1	—	—	—	0.71	0.504		
	PBC2	5.38	0.748	0.073	10.318	<.001	0.605	0.366		
	PBC3	5.015	0.935	0.092	10.178	<.001	0.596	0.355		
**Subjective norms (SN)**									0.758	0.512
	SN1	5.16	1	—	—	—	0.704	0.496		
	SN2	5.2	1.122	0.085	13.162	<.001	0.764	0.584		
	SN3	5.023	1.009	0.085	11.884	<.001	0.675	0.456		
**Trust (TR)**									0.691	0.429
	TR1	5.359	1	—	—	—	0.583	0.34		
	TR2	4.595	1.697	0.166	10.228	<.001	0.732	0.536		
	TR3	4.975	1.349	0.141	9.551	<.001	0.642	0.412		
**Resistance bias (RB)**									0.767	0.524
	RB1	2.319	1	—	—	—	0.683	0.466		
	RB2	2.479	1.368	0.109	12.567	<.001	0.762	0.581		
	RB3	2.259	1.133	0.093	12.123	<.001	0.724	0.524		
**Eye health consciousness (EHC)**									0.766	0.625
	EHC1	6.051	1	—	—	—	0.876	0.767		
	EHC2	5.724	0.859	0.136	6.317	<.001	0.694	0.482		
**Perceived risks (PR)**									0.837	0.461
	PR1	3.962	1	—	—	—	0.711	0.506		
	PR2	3.979	0.932	0.073	12.814	<.001	0.639	0.408		
	PR3	3.804	1.081	0.075	14.468	<.001	0.738	0.545		
	PR4	3.308	0.968	0.081	12.025	<.001	0.621	0.386		
	PR5	4.217	1.089	0.082	13.34	<.001	0.705	0.497		
	PR6	3.544	0.931	0.075	12.483	<.001	0.654	0.428		
**Intention to use (IU)**									0.753	0.506
	IU1	4.977	1	—	—	—	0.743	0.552		
	IU2	5.251	0.999	0.069	14.436	<.001	0.769	0.591		
	IU3	5.348	0.779	0.069	11.361	<.001	0.612	0.375		

^a^Unstd: unstandardized factor loadings.

^b^STD: standardized factor loadings.

^c^SMC: square multiple correlations.

^d^CR: composite reliability.

^e^AVE: average variance extracted.

^f^Not applicable.

**Table 7:** Discriminant validity.

Constructs	AVE^a^	PU	PR	IU	RB	EHC	SN	PEOU	PBC	TR
Perceived usefulness (PU)	0.431	*0.657^b^*	—^c^	—	—	—	—	—	—	—
Perceived risks (PR)	0.462	–0.266	*0.680*	—	—	—	—	—	—	—
Intention to use (IU)	0.506	0.458	–0.364	*0.711*	—	—	—	—	—	—
Resistance bias (RB)	0.524	–0.318	0.424	–0.374	*0.724*	—	—	—	—	—
Eye health consciousness (EHC)	0.625	0.309	–0.179	0.277	–0.272	*0.791*	—	—	—	—
Subjective norms (SN)	0.512	0.432	–0.289	0.471	–0.236	0.179	*0.716*	—	—	—
Perceived ease of use (PEOU)	0.516	0.430	–0.223	0.324	–0.244	0.171	0.296	*0.718*	—	—
Perceived behavioral control (PBC)	0.408	0.383	–0.360	0.374	–0.116	0.181	0.453	0.380	*0.639*	—
Trust (TR)	0.429	0.343	–0.332	0.422	–0.152	0.126	0.458	0.247	0.411	*0.655*

^a^AVE: average variance extracted.

^b^The items on the diagonal in italics represent the square root of the AVE; off-diagonal elements are the correlation estimates.

^c^Not applicable.

In Table 7, the square roots of the AVE values (the italic numbers on the diagonal) are higher than the numbers in the off-diagonal direction (correlations between a particular construct in the same column and other constructs in different rows) in the corresponding columns, indicating that the discriminant validity of all constructs meets the criteria of Fornell and Larcker [76].

### Structural Model Analysis

Table 8 presents the model fit indicators with their respective criteria: (1) the standardized root mean square residual is 0.057, smaller than 0.08, (2) the comparative fit index is 0.915, greater than 0.90, and (3) the root mean squared error of approximation is 0.049, also smaller than 0.08. The model fit indicators shown in Table 8 satisfy most of the criteria and the combination rule [77], indicating that the hypothesized model has a good fit to the data.

**Figure 2:**
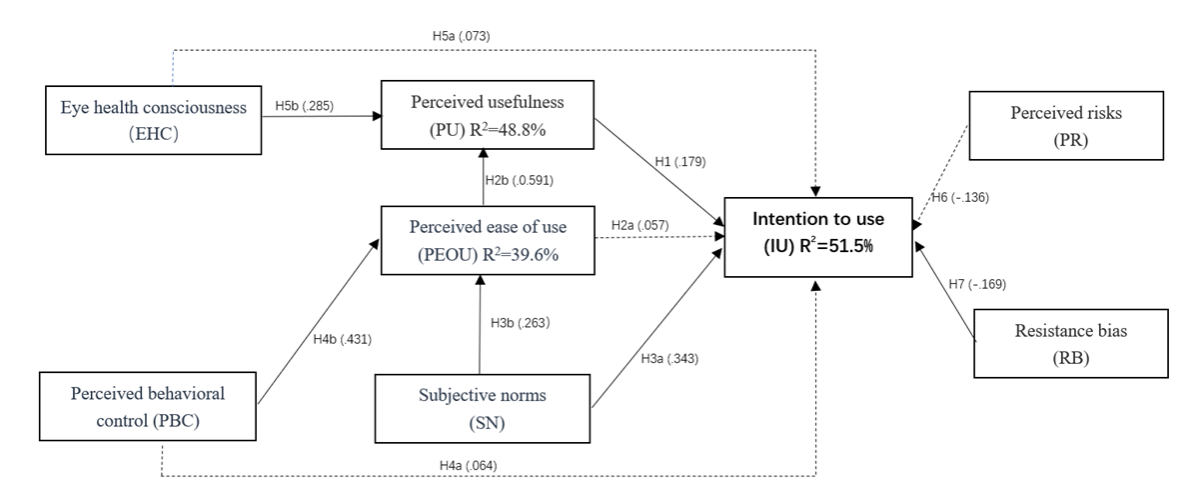
Estimates of regression analysis. Note: Solid line indicates a significant path and dotted line indicates a nonsignificant path.

Figure 2 shows the graphic description, and Table 9 shows the numerical results of the path coefficients. IU is significantly affected by SN (beta=.408; *P*<.001), PU (beta=.336; *P*=.03), and RB (beta=–.237; *P*=.02). PEOU (beta=.050; *P*=.59), EHC (beta=.077; *P*=.25), PBC (beta=–.066; *P*=.52) and PR (beta=–.133; *P*=.01) do not significantly affect IU. PEOU is significantly affected by PBC (beta=.506; *P*<.001) and SN (beta=.354; *P*=.002). PU is significantly affected by EHC (beta=.159; *P*<.001) and PEOU (beta=.279; *P*<.001).

R^2^ was calculated to access the validity of the research model. As Table 9 and Figure 2 show, 51.5% of IU can be explained by PU, SN, PEOU, RB, and PR constructs; 48.8% of PU can be explained by the EHC and PEOU constructs; and 39.6% of PEOU can be explained by the SN and PBC constructs.

**Table 8:** Model fit of the research model.

Model fit	Criteria	Model fit of research model
χ^2a^	The smaller the better	755.629
*df*	The larger the better	356.00
Normed chi-square (χ^2^/*df*)	1<χ^2^/*df*<3	2.123
RMSEA^b^	<0.08	0.049
SRMR^c^	<0.08	0.057
CFI^d^	>0.9	0.915
GFI^e^	>0.9	0.896
AGFI^f^	>0.8	0.873

^a^χ^2^: chi-square.

^b^RMSEA: root mean squared error of approximation.

^c^SRMR: standardized root mean square residual.

^d^CFI: comparative fit index.

^e^GFI: goodness-of-fit index.

^f^AGFI: adjusted goodness-of-fit index.

**Table 9:** Regression coefficient.

Dependent variables and hypothesis (H)		Unstd^a^	SE	*T* value	*P* value	Std^b^	Supported	R^2^
**IU^c^**								0.515
	IU←PU^d^ (H1)	0.336	0.151	2.219	.03	0.179	✓	
	IU←PEOU^e^ (H2a)	0.05	0.093	0.544	.59	0.057	X	
	IU←SN^f^ (H3a)	0.408	0.098	4.146	<.001	0.343	✓	
	IU←PR^g^ (H6)	–0.124	0.066	–1.875	.06	–0.136	X	
	IU←RB^h^ (H7)	–0.237	0.102	–2.328	.02	–0.169	✓	
	IU←EHC (H5a)	0.077	0.066	1.156	.25	0.073	X	
	IU←PBC (H4a)	0.066	0.104	0.64	.52	0.064	X	
**PU**								0.488
	PU←EHC^i^ (H5b)	0.159	0.031	5.14	<.001	0.285	✓	
	PU←PEOU (H2b)	0.279	0.034	8.128	<.001	0.591	✓	
**PEOU**								0.396
	PEOU←SN (H3b)	0.354	0.116	3.051	.002	0.263	✓	
	PEOU←PBC^j^ (H4b)	0.506	0.11	4.59	<.001	0.431	✓	

^a^Unstd: unstandardized factor loadings.

^b^Std: standardized factor loadings.

^c^IU: intention to use.

^d^PU: perceived usefulness.

^e^PEOU: perceived ease of use.

^f^SN: subjective norms.

^g^PR: perceived risks

^h^RB: resistance bias.

^i^EHC: eye health consciousness.

^j^PBC: perceived behavioral control.

### Analysis of Mediation Effects

Bias-corrected bootstrapping mediation analysis (5000 iterations) was used to examine the indirect effects (Table 10).

PU fully mediates the effect of EHC on IU (95% CI 0.04 to 0.1361). PEOU and PU fully mediate the effect of PBC on IU (95% CI 0.01 to 0.2322), whereas PEOU partially mediates the effect of PBC on PU (95% CI 0.057 to 0.2697). PEOU and PU do not mediate the effect of SN on IU (95% CI –0.0011 to 0.2000), whereas PEOU partially mediates the effect of SN on PU (95% CI 0.0004 to 0.2517). PU also fully mediates the effect of PEOU on IU (95% CI 0.005 to 0.2398).

### Analysis of Moderation Effect

**Figure 3:**
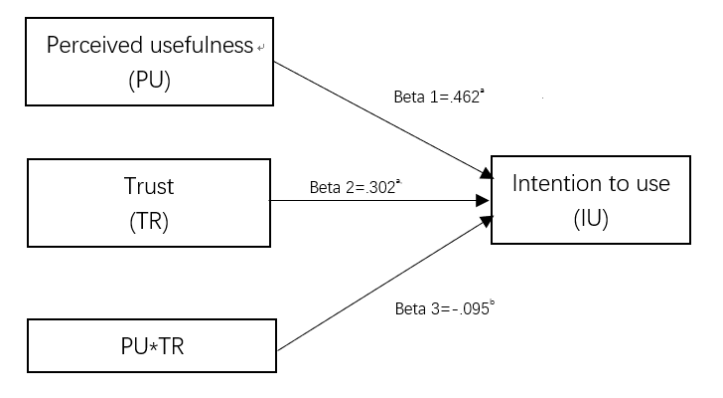
Trust moderates the effect of PU on IU. a *P*<.01; b *P*<.05.

In Figure 3 and Table 11, the trust moderates the effect of PU on IU (beta=–.0.095; *P*=.049), where the effect of PU on UI is stronger for the users with low trust compared with those with high trust.

**Table 10:** Analysis of indirect effects.

Paths relationship	Direct effect (95% CI)			Indirect effect (95% CI)			Results
	Effect	LLCI^a^	ULCI^b^	Effect	LLCI	ULCI	
EHC^c^→PU^d^→IU^e^	0.0765	–0.0636	0.2443	0.053	0.004	0.1361	Fully
PBC^f^→PEOU^g^→PU→IU	0.0663	–0.202	0.3133	0.073	0.001	0.2322	Fully
PBC→PEOU→IU	0.0663	0.202	0.3133	0.073	0.001	0.2322	Fully
PEOU→PU→IU	0.0504	–0.1868	0.3059	0.094	0.005	0.2398	Fully
PBC→PEOU→PU	0	0	0	0.141	0.057	0.2697	Partial
SN^h^→PEOU→PU	0	0	0	0.099	0.0004	0.2517	Partial
SN→PEOU→PU→IU	0.4083	0.1768	0.6509	0.051	–0.0011	0.2	No
SN→PEOU→IU	0.4083	0.1768	0.6509	0.051	–0.0011	0.2	No

^a^LLCI: lower limit confidence interval.

^b^ULCI: upper limit confidence interval.

^c^EHC: eye health consciousness.

^d^PU: perceived usefulness.

^e^IU: intention to use.

^f^PBC: perceived behavioral control.

^g^PEOU: perceived ease of use.

^h^SN: subjective norms.

**Table 11:** Moderation analysis.

Dependent variable, independent variable		Unstd^a^	Std^b^	SE	*P* value	Bootstrap 1000 times, bias-corrected 95% CI
**Intention to use**						
	Perceived usefulness	0.934	0.462	0.237	<.001	0.4691 to 1.3997
	Trust	0.857	0.302	0.287	.003	0.2937 to 1.4209
	Perceived usefulness×Trust	–0.095	–0.095	0.048	.049	–0.1897 to –0.0001

^a^Unstd: unstandardized factor loading.

^b^Std: standardized factor loadings

## Discussion

### Principal Findings

This study investigated the relationships between factors that affect the adoption of ophthalmic AI devices for eye health management. The research model was developed using relevant theories of technology acceptance, including TAM, TPB, HBM, DFT, SQB, and Trust to fit AI applications in particular health care scenarios in China.

There are 4 principal findings: (1) SN plays a more important role than PU through both direct and indirect paths; (2) RB of new technology reduces public IU of ophthalmic AI, whereas PR does not have an effect on public IU; (3) EHC and PBC have an indirect positive effect on the IU of AI through the mediators PU and PEOU; and (4) trust moderates the effect of PU on IU. The results are discussed in detail below.

#### Subjective Norms Play a Much More Important Role in Artificial Intelligence Adoption Than Perceived Usefulness

As many studies have discussed, PU, PEOU, SN, and PBC significantly influence IU [31,32,53]. However, the function of SN differs among cultures. Some studies have found no significant effects [29,78], whereas others have reported the opposite result [59]. In our study, SN was the most important predictor of IU, whose direct effect on IU was much stronger than that of PU. It also had a significant positive effect on PU through PEOU. These results indicate that, in China, when individuals encounter new technologies such as ophthalmic AI devices, public perceptions about usefulness, ease of use, and IU are likely to be influenced by their significant others (the items of the SN construct) such as close friends and relatives, colleagues and peers, and superiors or leaders in their work teams. This phenomenon could be linked to a crowd mentality (following the group’s actions), collectivist culture (prioritizing a group over the individual), authoritarianism (follow the rule of team leaders), and Confucianism (conforming to prescribed relationship roles and avoiding transgression) in China.

Furthermore, an interesting finding was that PEOU did not have a significant direct effect on IU, so H2 was not supported. This finding means that the public’s IU of ophthalmic AI was not influenced by perceptions of how easy these technologies would be to use. However, the average score of the construct PEOU was high, with a value of 5.739 out of 7. One possible explanation is that because ophthalmic AI devices are newly developed products, the public might perceive them as *intelligent* and believe that they should be convenient and easy to use. Although the direct effect of PEOU on IU was not significant, PEOU did have a strong effect on PU, confirming most TAM theories. Therefore, if someone whose opinion was important to participants suggested that they try the devices (SN), and the participants then realized the value or usefulness (PU) of the devices, participants’ IU would be high.

#### Resistance Bias Reduces Public Intention to Use Ophthalmic Artificial Intelligence Whereas Perceived Risks Do Not Have an Effect on It

In most research on the Dual Factor Theory, PR negatively affected the public’s IU. However, in our ophthalmic AI case, PR does not affect public’s IU. This finding is in line with the Chinese context where people do not perceive risk of blindness as an acute threat and owing to the fact that the general population of China does not strongly prioritize privacy [79]. The low mean score of the 6 items of the PR construct (3.802 out of 7) reflects the public’s lack of awareness of health risks and protection of health information and privacy. These results were also confirmed by our qualitative study that people are accustomed to providing key personal information when registering on an app or receiving nuisance calls.

We also integrated a new construct, RB, and verified its reliability and validity in our model, improving our understanding of negative factors involved in health care technology acceptance. Our results confirmed the SQB theory. People might reject ophthalmic AI devices owing to unfamiliarity, regret avoidance, or past experiences with new technology products. This resistance reflects many people’s natural preference to continue with traditional approaches to health management. This finding matches observations about Chinese patients’ acceptance of mobile phone health technology and mobile health services for chronic disease management that these inhibitors had a negative effect on behavioral intention [31,57].

#### Eye Health Consciousness and Perceived Behavioral Control Have an Indirect Positive Effect Via the Mediators Perceived Usefulness and Perceived Ease of Use

Previous studies of health behavior based on the theories of HBM have found that PHT (similar to EHC) has both direct and indirect effects on IU [31,36]. In our study, EHC had a significant positive influence on PU and an indirect influence on IU via PU. However, EHC had no significant direct effect on IU, which contrasts with the findings of Dou about Chinese patients’ acceptance of mobile phone health technology [31] but is consistent with the work of Kim about consumers’ health behavior IU of health information technology [36]. Our findings could indicate that although people are conscious about their eye health and perceive health threats even without eye screening, they will assess the usefulness or function of new AI devices before switching from traditional face-to-face eye examination by ophthalmologists.

Many studies have found that perceived behavioral control has a significant direct and indirect influence on IU [53,57]. We found that it had no significant impact on IU, consistent with the meta-analysis of factors influencing mobile health service adoption [32], but in contrast with research on health professionals’ adoption of health clouds [51] and physicians’ acceptance of electronic medical record exchange [53]. Our findings could result from the different roles of general public and medical staff, as most health-related procedures in previous studies were conducted by medical staff, whose behavioral controllability of new developed devices was a more important concern during the manipulation process. However, we found that PBC had an indirect effect on IU through PEOU and PU. This indicates that unlike other health-related technologies studied, as emerging products, ophthalmic AI devices need to be convenient and useful to ensure the perception of behavioral controllability. The high average score of PBC items (5.005 out of 7) also shows that if the public perceived these devices as easy to use and useful, automanipulation of screening devices and self-management of eye screening could be achieved.

#### Moderation Effect of Trust

Previous studies have treated trust as a variable that affects IU directly or indirectly [44,52]. Few studies have discussed whether it could be a moderator. In China, in the context of unbalanced medical resource distribution and distrust between doctors and patients, this construct could play a more complicated role [66,68,80]. Our finding confirmed that this construct is a moderator, as trust had a significant moderation effect (beta=–.095; *P*=.049) on the path from PU to IU. The public’s trust in the emerging technology and medical staff negatively moderated the influence of PU on IU. Participants with high trust in AI might have high expectations for AI in health care and thus might require greater PU before they would be willing to try the AI devices. Alternatively, participants with low trust in AI might have low expectations and require less PU before trying to use them. In light of the generally distrustful relationship between the public and medical staff in China, stakeholders such as doctors and AI suppliers should avoid making misleading or over-exaggerated claims in the promotion of AI health care products.

As the beta value was negative, the more the public trust AI devices, the lower the effect of PU on IU. In other words, if we improve people’s beliefs and confidence about AI products, they will use these devices even if these devices are not as useful as they could be. In our study, the average score for the 3 trust items was 4.976 out of 7. Together with the low factor loading of PU on IU, in the Chinese context, the influence of PU was small. We interpreted this effect to be moderated by trust.

### Comparison With Prior Work and Strengths of This Study

This study contributes to the AI health care literature in several ways:

1. This study was the first empirical study to examine the positive and negative factors that influence public acceptance of emerging AI devices in real clinical scenarios in China. As the model fit and R^2^ values are high, our model can predict the Chinese public’s IU of such AI devices.
2. We integrated 1 inhibitor of RB to modify the SQB theory to fit Chinese people’s thinking style and language customs, and this showed both good convergence and discriminant validity.
3. We introduced trust as a moderator in the Chinese social context to reflect the health care context, and the results confirmed that trust had a moderation effect on the path from PU to IU.
4. SN has the greatest effect on the IU of AI devices. This finding differs from most studies of new health care technology acceptance and could reflect the culture, regulations, or rules in the Chinese social context.
5. PR does not significantly affect public’s IU as participants were not aware of the protection of personal privacy and health information.

### Implications for Practice

Researchers are only just beginning to assess how we might improve medicine using neural networks, and we will not know how well AI can predict key outcomes in health care settings without “robust validation in prospective, real-world clinical environments, with rigorous statistical methodology and analysis” [9]. As our data show, PU does not play as important a role as expected, and the following strategies could be a cost-effective way to improve public acceptance of AI devices and promote AI products in the era of narrow AI:

1. Enhance public trust in AI, avoiding misleading or exaggerated claims that might affect public perceptions of the function of AI in health care.
2. Expand the influence of SN through health communication campaigns in communities and workplaces, focusing on significant others in people’s social circles such as superiors, public opinion leaders, and close friends.
3. Educate the public’s knowledge and consciousness of the accuracy, effectiveness, safety, and privacy of AI devices and expedite legislation on AI to protect human rights.

### Limitations and Future Research

Our nationwide study included people of all ages, from students to elders, which indicated good external validity. It was more cost-effective to recruit participants nationally from the internet rather than by traditional means, and this method was more suitable in the AI context because it provided real-time reports and feedback for target users. However, as the sample was collected through mobile devices or websites, the proportion of participants aged above 50 years was relatively low. This age distribution could reflect the fact that older people are less likely to use mobile devices owing to poor vision or motor abilities or RTC [58]. If automated or self-management procedures with AI products require good mobile or digital skills, older people might not be appropriate target users. In future studies, we might introduce age as a moderator to evaluate its interference effect. When designing or promoting an AI device, we should consider its practical utility for older generations, as they are the main screening population in primary care projects. Moreover, medical staff such as hospital leaders, physicians, and nurses would be the main users of these devices, so their views on AI are very important. We will conduct further research on their intention to adopt and manipulate ophthalmic AI devices in real clinical scenarios.

### Conclusions

Our study used the SEM method to explore the complex relationships between factors that influence public acceptance and IU of ophthalmic AI devices, as applied to real clinical scenarios in China. Positive factors such as SN played a more important and complex role than predicted, alongside people’s EHC and PBC, whereas the inhibiting factor, RB, had a direct negative effect on adoption of AI devices. The new integrated inhibitor of RB fits Chinese people’s thinking style and language customs and showed both good convergence and discriminant validity. PR does not significantly affect public’s IU as they were not aware of the protection of personal privacy and health information. Furthermore, we found that trust had a moderation effect on the path from PU to IU. This integrated model, incorporating Chinese cultural and social contexts, demonstrated a good fit and explanatory power with high R^2^ and could be used to explore other AI health care areas such as chronic disease screening and monitoring, especially for diabetes, hypertension, and cancer management.

## Appendix

Multimedia Appendix 1 Answers for CHERRIES.

## Abbreviations

AI: artificial intelligence
AVE: average variance extracted
CHC: community health center
CR: composite reliability
DFT: Dual Factor Theory
DR: diabetic retinopathy
EHC: eye health consciousness
HBM: health belief model
IS: incumbent system
IU: intention to use
PBC: perceived behavioral control
PEC: primary eye care
PEOU: perceived ease of use
PHT: perceived health threat
PR: perceived risks
PU: perceived usefulness
RB: resistance bias
RTC: resistance to change
SEM: Structural equation modeling
SN: subjective norms
SQB: status quo bias
TAM: Technology Acceptance Model
TPB: Theory of Planned Behavior
TR: trust
UTAUT: Unified Theory of Acceptance and Use of Technology
