# Supplementary material for: Psychosocial Factors Affecting Artificial Intelligence Adoption in Health Care in China: Cross-Sectional Study
Source: J Med Internet Res. 2019 Oct 17;21(10):e14316. doi: 10.2196/14316 (PMC6913088; doi:10.2196/14316)
Supplement: Multimedia Appendix 1 [file jmir_v21i10e14316_app1.pdf]

Item category	Checklist Item	Explanation
Design	Describe survey design	1. Target population are Chinese mobile phone users nationwide using Wechat for communication. They are the main potential users of ophthalmic AI devices.<br>2. Sampling frame: We conducted a randomized sampling using an online-survey company's (Wenjuanxing) sample service, which has an online sample database (Shoujiang Website) with nearly 2.6 million people around China, covering different ages and occupations. The technician of this company sent invitation in certain time each day randomly using their own developed system.
IRB (Institutional Review Board) approval and informed consent process	IRB approval	Our study has been approved by The Ethics Committee of the Zhongshan Ophthalmic Center, Sun Yat-Sen University (SYSU).
	Informed consent process	On the first page of our online questionnaire, we told the participants the length of time of the survey, that the investigators are from SYSU and that the purpose of this study was to investigate the factors influencing Chinese public acceptance of ophthalmic AI devices. Participants were told that all data were stored anonymously in a cloud-based server by the PI of this study, and only researchers on the study team can have access to the data. All the participants have right to delete their data anytime without any reasons.
	Data protection	1. All personal information was anonymous and we cannot know the real name, or phone number of any participants. What we know were IP location, gender, career type and age.<br>2. All the data were collected and stored by researchers and kept in the PI's laptop, which are password protected and only researchers in our team have this password that can access them with her permission.
Development and pre-testing	Development and testing	1. We searched related literatures and review them carefully and selected 9 latent variables with 12 hypothesis routine.<br>2. 9 latent variables were measured by 32 questionnaire items. All items were sourced from the relevant literature related to consumer technology acceptance research, with some changes to fit the ophthalmic AI context (Table 1).<br>3. Items in English were translated into Chinese by 1 researcher and checked by 3 other researchers, and 1 researcher then back translated the items into English to check if the original meaning was retained. All researchers are fluent in both Chinese and English.<br>4. All items were measured on 7-point Likert scales ranging from (1) strongly disagree to (7) strongly agree.<br>5. We asked 1 experts of SEM, 1 experts of public health and 1 expert of medical AI to evaluate our items and variables of our questionnaire, modifying language and sequence of lists to fit Chinese rhetoric.
**Recruitment process**	Open survey versus closed	It is a closed survey only open to the participants from the sample database (Shoujiang website) of the

**and description of the sample having access to the questionnaire**	survey	Wenjuanxing company.
	Contact mode	The company sent participants a Wechat message with the link of our questionnaire during the data collection days. Surveys could be completed by potential participants using Wechat.
	Advertising the survey	In order to advertise the survey, Shoujiang website sent a message to users on the platform through a Wechat message stating: “the link of the questionnaire, credits 400”. After they clicked into the link, the title and informed consent were displayed. The participants received 400 credits after their completed questionnaire was confirmed to be valid. 100 credits will be exchanged into 1 RMB. So the incentives of credits would be the advertisement.
**Survey administration**	Type of e-survey	Our survey was posted on a website through the platform (Shoujiang Website) of Wenjuanxing and participants could access it from Wechat.
	Context	Wenjuanxing is the most popular survey company in China and nearly every mobile phone user who has Wechat had experiences of answering surveys through Wenjuanxing. It can select participants who are mobile phone users from different regions of China randomly, which is our target population.
	Voluntary	It was a voluntary survey that participant could neglect the Wechat message if they do not want to answer the questionnaire.
	Incentives	Participants would receive 4 yuan after their questionnaire was evaluated valid. 100 credits could be exchange int 1 yuan and the total credits was shown in the Wechat message.
	Time/Date	January 20 to 24, 2019
	Randomization of items or questionnaires	It is categorized logically so that participants will be easy to understand and answer. We did not provide the direct purpose of each section so that they won’t guess the “right” answer and avoid the potential bias.
	Adaptive questioning	No adaptive questions were used.
	Number of Items	First page was the introduction and informed consent of this survey. The page two to four were the main content of questionnaire and we divided 32 items into 6 sections. Each page had two sections. The fifth page contained the rest items for basic demographic information of the participants.
	Number of screens (pages)	Five.
	Completeness check	All the questions were mandatory items. As we used Likert scale, the middle option was “I’m not sure”, which is similar to the “non-response option” such as “not applicable” or “rather not say” as CHERRIES required.
	Review step	Respondents were able to review and change their answers through a Back button on the end of each page.
**Response rates**	Unique site visitor	In this sample database, each participant has only one account name which was defined based on IP addresses and linked to their ID number with authentication.

	View rate (Ratio of unique survey visitors/unique site visitors)	This metric was not calculated by the website.
	Participation rate	The number of people who filled in the first survey page and agreed to participate (including both completed or uncompleted questionnaires) was 732. And the number of visitors who visit the first page of the survey was 925. So that the participation rate or recruitment rate was 732/925=79.1%.
	Completeness rate	474 submitted the last questionnaire page and met the criteria and were used for the SEM analysis. The number of people who agreed to participate (or submitted the first survey page) was 732. Because "completion" has not involved leaving questionnaire items blank, so that we use a measure of completeness rate, which was 474/732=64.8%.
**Preventing multiple entries from the same individual**	Cookies used	No.
	IP check	No.
	Log file analysis	Participants had their only account and bounded with his/her Wechat account. If a participant wants to get an incentive by answering a questionnaire, he/she has to authenticate with their real names and at least one bank account, which is linked with his/her unique ID number.
	Registration	When potential participants login to Wechat, they would receive a message saying that there is a questionnaire waiting to be answered and an incentive of about four yuan after completion and valid verification. If they want to receive the incentive, they must authenticate with their real names and at least one bank account, and also must be linked to his/her unique ID number. Even if the participants had more than one account, they would not enter the questionnaire and would be told that you have already answered this survey and the survey would not be shown to them twice.
**Analysis**	Handling of incomplete questionnaires	Only completed questionnaires were analyzed.
	Questionnaires submitted with an atypical timestamp	In total, the survey instrument was five pages long. Assuming participants took one minute per page, ten seconds per item, as well as time to complete informed consent and demographics, we set a criterion of 300 seconds for minimum length to complete the questionnaire. We excluded questionnaires submitted in under 300 seconds.
	Statistical correction	No methods such as weighting of items or propensity scores have been used because we did not analyze the non-representative samples.
